# A Mixed-Methods Approach to the Examination of Cancer Treatment Preferences Among Older Latino Adults

**DOI:** 10.1093/geroni/igab046.089

**Published:** 2021-12-17

**Authors:** Iraida Carrion, Malinee Neelamegam, Tania Estape

**Affiliations:** 1 University of South Florida, Tampa, Florida, United States; 2 Department of Epidemiology of Microbial Diseases Yale School of Public Health, New Haven, Connecticut, United States; 3 FEFOC, Fundacion Contra El Cáncer, Barcelona, Catalonia, Spain

## Abstract

Given the growing population of Latino immigrants 60 years and older and the current lack of relevant data, there is an urgent need to understand this population’s cancer treatment preferences to ensure effective interventions and psychosocial care. A study comprising 200 surveys with areas focused on cancer knowledge, attitudes, prevention, early diagnosis, and treatment was developed and administered in Spanish. The survey included a qualitative component consisting of open-ended questions. The mixed-method study gathered quantitative data regarding treatment preferences as well as the voices of older Latino men and women with a history of cancer, including their cancer treatment trajectory. Additionally, the survey data highlighted the lack of knowledge regarding available cancer treatments. The findings suggest that, while older Latino/as have knowledge about the causes of cancer, they lack knowledge regarding cancer diagnoses, which could potentially cause them to avoid treatment.

